# Pre-pregnancy weight loss associations with prenatal and postpartum mental health conditions: A retrospective cohort study

**DOI:** 10.21203/rs.3.rs-3232490/v1

**Published:** 2023-09-05

**Authors:** Megan Ferber, Timothy Chrusciel, Sophia Cantwell, Joanne Salas, Kara M. Christopher

**Affiliations:** Saint Louis University; Saint Louis University; Saint Louis University; Saint Louis University; Saint Louis University

**Keywords:** depression, anxiety, weight loss, pregnancy

## Abstract

**Background:**

Obesity is a risk factor for adverse outcomes during and following pregnancy. Most women are advised to lose weight prior to becoming pregnant, to help alleviate complications including prenatal and postpartum depression and anxiety. Yet, no studies have examined how the process of losing weight prior to pregnancy interacts with the development of prenatal and postpartum mental health disorders.The objective of the study was to determine if women with pre-pregnancy weight loss (≥10%) vs. those who do not, in the two years prior to pregnancy, have a lower risk for new onset prenatal and postpartum mental health conditions

**Methods:**

This retrospective cohort study used data from the Virtual Data Warehouse of a large Midwestern, U.S. based hospital system. The final sample consisted of 6,085 female patients of reproductive age that had given birth between 10/1/2011–6/30/2020 and had two recorded weights in the year prior to conception. Univariate analysis between weight loss and outcome variables (pre-natal and post-partum depression and anxiety) and multivariate analysis using logistic regression was conducted for variable significant on univariate analysis.

**Results:**

On univariate analysis, women with pre-pregnancy weight loss had increased odds of post-partum depression (OR=1.47, 95%CI=1.03–2.10), though decreased odds of prenatal anxiety (OR=0.59, 95% CI 0.33–0.90). After controlling for confounders in the multivariate analysis, there was not a significant difference in the odds of post-partum depression; however, women who lost weight had approximately half the odds of having prenatal anxiety than those who did not lose weight (OR=0.54, 95%CI=0.33–0.90).

**Discussion:**

The experience of achieving weight loss prior to pregnancy may foster a sense of agency within pregnant women, helping them to reduce their experience of pre-natal anxiety. Providers could engage in patient conversations around weight loss and mental health management in a strengths-based framework to continue to foster this sense of agency.

**Conclusion:**

Anxiety and depression were uniquely related to pre-pregnancy weight loss. Pre-pregnancy weight loss was associated with lower odds of prenatal anxiety and higher odds of postpartum depression. These results highlight the need for real world examination of pre-conception treatment recommendations and their association with non-physical health-based outcomes.

## Introduction

Rates of overweight (body mass index (BMI) = 25.0–29.9kg/m^2^) and obesity (BMI ≥ 30.0kg/m^2^) among adult American women have continuously increased for the past 20 years, with 41.9% experiencing obesity in 2018 (Ogden et al., 2020). Obesity is a risk factor for adverse outcomes in the 85% of women who become pregnant by age 44 (Martinez et al., 2018). Most women with overweight or obesity are advised to lose weight prior to becoming pregnant to help alleviate several pregnancy and postpartum complications (ACOG, 2021). One of these complications is the development of prenatal and postpartum mental health disorders, including depression and anxiety (Jackson et al., 2014). Around 10–25% of mothers will experience depression during pregnancy (Accortt et al., 2015) and 10–15% in the postpartum period (Flynn, 2005) while 0.9%−22.7% of mothers will experience generalized anxiety disorder during pregnancy (Viswasam et al., 2019) and 4.4–8.5% postpartum (Ross et al., 2006). Mothers who had overweight or obesity at time of pregnancy appear to have higher risk for the development of postpartum depression and anxiety compared to their normal weight counterparts (Dachew et al., 2020). However, it remains unknown how the process of losing weight prior to pregnancy is associated with the development of prenatal and postpartum depression and anxiety among these women.

In the general population, losing weight, defined as losing at least 5–10% of one’s body weight (Wadden et al., 2004), has produced mixed results in terms of changes in mental health symptoms. Some evidence indicates weight loss is associated with improved depressive (Busch et al., 2013) and anxiety symptoms (Rodriguez-Lozada et al., 2019) while others have found that weight loss was associated with increased depression symptoms (Fabricatore et al., 2011) and no association with anxiety (de Wit et al., 2015). However, no studies have examined how the process of losing weight prior to pregnancy interacts with the development of prenatal and postpartum mental health disorders.

Women do attempt to make lifestyle changes prior to becoming pregnant. Previous scholars have found that attempting to decrease alcohol use and improve dietary quality as the most frequently endorsed improvements women report (Poels, et al., 2017) and that there were no differences in these behavior changes based on weight status (Stephenson, et al., 2017). These improvements could result in changes in one’s body weight. One United Kingdom-based study conducted found that roughly 18% of women with unhealthy body weight prior to conception achieved a healthier weight status once pregnant (Stephenson, et al., 2017). While previous studies have linked improved maternal and fetal physical health outcomes (Stephenson, et al., 2017), previous studies have not examined their associations with mental health outcomes, especially amongst those who have achieved a significant amount of weight loss.

Using data from a large, Midwestern hospital system, this study aims to determine if patients who experience successful weight loss (losing ≥ 10% of one’s body weight) vs. those who do not, in the two years prior to pregnancy, have a lower risk for new onset prenatal and postpartum mental health conditions. The results of this study seek to identify specific time points (prenatal or postpartum) that may be most vulnerable to psychopathology in a large, population level dataset. By identifying these individuals and timepoints, empirically-supported interventions can be implemented and tested for efficacy in a targeted manner.

## Methods

### Sample and Procedures

This retrospective cohort study used de-identified medical record data from the XXXX University-XXX HealthCare System’s Virtual Data Warehouse (VDW). The VDW captures clinical encounter data starting in 1/1/2008 from academic and non-academic ambulatory and inpatient settings in a Mid-western, multi-state health care system. The health care system covers rural and urban locations from the southern half of Wisconsin, Southern Illinois, the St. Louis, Missouri metropolitan area, mid-Missouri, and the Oklahoma City, Oklahoma metropolitan areas. Research use of the VDW has received ethical approval from the Saint Louis University’s Institutional Review Board; thus informed consent was waived for the current study.

The VDW includes patients from birth to > 90 years of age who have private or public health insurance as well as uninsured who utilized health care services within the XXX Health system. The VDW is updated monthly and includes over 5 million unique patients who had at least one encounter in the health care system since 1/1/2008. As a member site of the Health Care Systems Research Network (www.hcsrn.org), VDW variables are defined according to HCSRN specifications. VDW variables are created from ICD-9 and ICD-10 codes, current procedural terminology (CPT) codes, pharmacy orders, laboratory orders and results, vital signs, provider type, clinic type, and demographics. The Saint Louis University Institutional review board deemed this work to be exempt from ethics approval as non-human subjects research because data were retrospective and de-identified.

**Figure 1:**
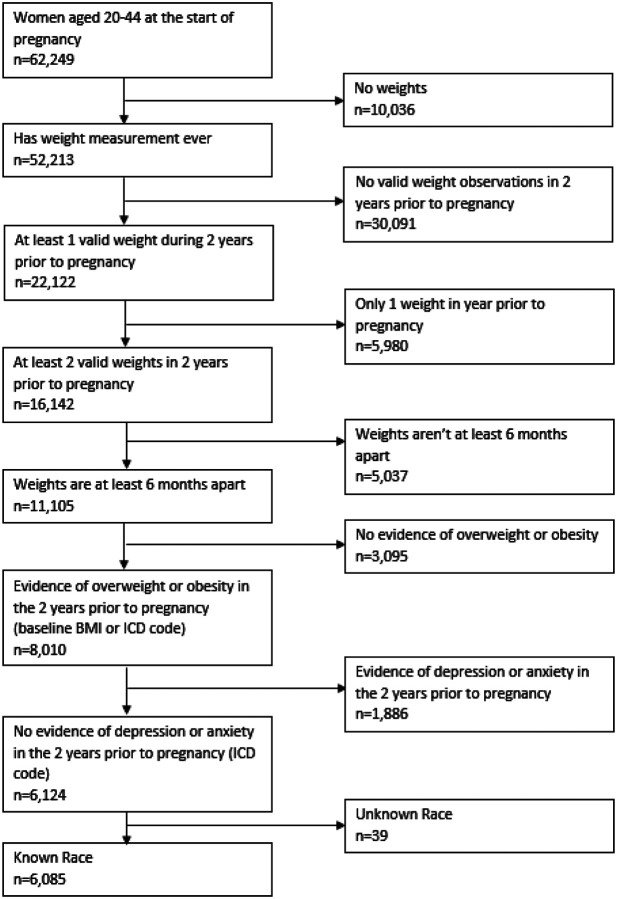


The primary inclusion criteria were female gender, live birth/delivery between 10/1/2011–6/30/2020, and age 20–44 at the start of pregnancy. This resulted in 62,249 eligible patients. We chose this date range because at the time the analysis was conducted (Fall, 2021), we wanted to have a year after birth to measure post-partum depression and anxiety, and this allowed ample lookback time for the start of pregnancy and the prenatal period. Live births were identified using CPT, ICD-9, and ICD-10 procedure codes. If no diagnosis of preterm birth was present, pregnancy start was calculated by subtracting 270 days from the date of delivery. If a diagnosis for preterm birth was present, pregnancy start was calculated by subtracting 245 days from the date of delivery. The post-partum observation period for outcomes was 1 year after delivery date. Patients were then excluded if insufficient weight information was available, patients were not overweight or obese in the 2 years prior to pregnancy, depression or anxiety were diagnosed in the 2 years prior to pregnancy, or race was unknown. This resulted in a final analytic sample of 6,085 patients. Variable definitions are shown in Appendix A and a detailed description of the sampling approach is shown in Fig. 1.

### Measures

Outcome variables: The outcomes of interest were prenatal and post-partum depression and anxiety, as defined by ICD-9/10 diagnostic codes. Anxiety was a composite of posttraumatic stress disorder, generalized anxiety disorder, panic disorder, obsessive compulsive disorder, social phobia, and anxiety disorder unspecified. Both anxiety and depression were defined based on the presence of ICD-9 or ICD-10 codes for the condition on 2 outpatient or 1 inpatient visits within the same observation period (pre-natal or post-partum). This algorithm has shown to have excellent agreement with manual chart abstraction and self-report (Solberg et al,. 2006; Frayne et al., 2010). Prenatal depression and anxiety were measured from pregnancy start date until birth. Post-partum outcomes were measured in the year after birth. See Appendix A for specific codes used.

Primary exposure: The primary exposure of interest was pre-pregnancy weight loss. Weight loss was calculated in the 2 years prior to pregnancy based on vital sign data (height and weight) by subtracting last available weight from the first available weight for each patient/participant/subject during this period. If there was less than 6 months between the first and last weight observation, this pregnancy was removed from analysis. The primary exposure was then categorized as loss of more than 10% of body weight (yes/no).

Covariates: Sociodemographic factors of age at pregnancy and race were assessed as potential confounders. Age was considered due to the expected increase in weight across time and race was included due to the disproportionate rates of obesity experienced by Black (Wang, et al., 2020 and Latina (Liu et.al., 2021) women within the United States.

### Analysis

Bivariate, unadjusted associations between weight loss (yes or no) and our outcomes were assessed using chi-square tests for categorical variables and independent sample t-tests for continuous variables. Multivariate analysis was performed using logistic regression for outcomes that showed significant effects in bivariate analysis. All analyses were conducted with SAS v9.4 at an alpha of 0.05.

## Results

As shown in Table 1, patients in this cohort on average were 28.4 years old. The patients (67.2%) were primarily white, followed by Black (26.0%). All four outcomes were observed in approximately 5% of the patients: 5.1% for prenatal depression, 4.5% for postpartum depression, 4.5% for prenatal anxiety, and 5.1% for postpartum anxiety. Patients that lost at least 10% of their body weight prior to pregnancy were significantly more likely to be white race (80.7% vs. 65.7%, *p* < 0.001). A significantly higher percentage of pre-pregnancy weight loss patients had postpartum depression (6.2% vs. 4.3%, p = 0.0341) and a significantly lower percentage had prenatal anxiety (2.8% vs. 4.7%, *p* = 0.0348). Pre-pregnancy weight loss was not significantly associated with prenatal depression or postpartum anxiety.

Table 2 describes the differences in weight loss, depression, and anxiety by race. Pre-pregnancy weight loss was observed in 11.8% of white patients, compared to 7.0% of Latina patients, 5.8% of Black patients, and 4.4% of patients of other races (*p* < 0.0001). Race was not associated with depression in the prenatal (*p* = 0.1411) or post-partum (p = 0.2430) periods. However, race was significantly associated with prenatal anxiety (*p* = 0.0035) and post-partum anxiety (*p* = 0.0006).

Logistic regression results for post-partum depression and prenatal anxiety are found in Tables 3 and 4. Pre-pregnancy weight loss was associated with 47% higher odds of post-partum depression prior to adjusting for confounders (OR 1.47; 95% CI 1.03–2.10). After adjusting for age and race, this association was similar in magnitude but no longer statistically significant (OR 1.43; 95% CI 1.00–2.04). Pre-pregnancy weight loss was associated with significantly lower odds of prenatal anxiety in the unadjusted logistic model (OR 0.59; 95% CI 0.36–0.97). After adjusting for age and race the magnitude of this association increased, and pre-pregnancy weight loss was associated with approximately half the odds of prenatal anxiety. (OR 0.54; 95% CI 0.33–0.90). In this model, being Black was also associated with decreased odds of prenatal anxiety compared to being white (OR 0.57; 95% CI 0.41–0.78). No regression was performed for the other two outcomes (prenatal depression and postpartum anxiety) because neither showed a significant association with pre-pregnancy weight loss during bivariate analysis.

## Discussion

In this study, we attempted to determine if there were notable differences in the incidence of prenatal and postpartum depression and/or anxiety among women who successfully lost 10% or more of their body weight in the 2 years prior to pregnancy compared to those who did not lose weight. Informed by previous studies (Busch et al., 2013; Cameron et al., 2019; Simon et al., 2010) that have demonstrated an improvement in depression or depressive symptoms in women after successful weight loss of 5% of body weight or more, we expected to see a similar outcome in our sample. However, we found that there was no difference in depression incidence in women who lost 10% of their weight in the two years prior to pregnancy after adjusting for age and race. Prior to adjustments, there was a positive association between pre-pregnancy weight loss and postpartum depression, which is in the opposite direction of previous findings. This may suggest that the post-partum period is a unique period for women experienced weight loss as they are experiencing a U-shaped trajectory, where they regain all the weight loss they lost and potentially more during pregnancy. This regain paired with the transition to parenthood could lead to the experience of postpartum depression. This may be a particularly risky period for young mothers and those of minoritized races, seeing as these adjustments in the models explained a majority of the variance.

Women who lost 10% of their weight in the two years prior to pregnancy had lower odds of experiencing prenatal anxiety after being adjusted for age and race. Anxiety has been inversely linked to a sense of control during the transition to parenthood, meaning the more a mother feels in control, the less anxiety she experiences (Busch et al., 2013; Cameron et al., 2019; Simon et al., 2010). It may be the by successfully losing weight prior to pregnancy, women gain a sense of control in their health that continues into pregnancy, thus reducing their odds of experiencing prenatal anxiety. The current study is unable to test for this mediation, which could be an important association to examine in future studies.

Further, Black mothers had lowered odds of experiencing prenatal anxiety. This result runs counter to past studies’ findings that Black mothers experience higher levels of prenatal anxiety compared to White and Latina mothers (Busch et al., 2013; Cameron et al., 2019; Simon et al., 2010). The sample examined in this study may be distinctive from these past studies as the current sample was restricted to those who were experiencing overweight or obesity prior to pregnancy. Past studies did not restrict their samples based on weight status. Cultural messaging regarding motherhood and body size varies based on the mother’s racial identity and may be create a unique experience for Black mothers compared to other mothers. Future studies could utilize an intersectional lens to examine the experience of mother’s racial identity, body size, and mental health.

This study does have some limitations, including the small sample size. Of the initial eligible sample size, less than 10% of the sample was eligible for inclusion primarily due to missing data. This also impacted the final analysis, as we were unable to stratify results by race as initially planned. There is also a chance of selection bias in our sample, the women who were included could differ from the rest of our population on any of the key variables. Additionally, this is an observational study and while we found associations between weight loss and new onset depression and anxiety, we cannot determine if the two are causally linked based on this study. Finally, because this is a secondary data analysis, we were limited to the variables and information contained in the VDW.

There are also strengths to our study. This is the first known study to examine impact of pre-pregnancy weight loss on new onset depression and anxiety. As more women attempt to lose weight prior to pregnancy through traditional or surgical methods, there is a need to care for the whole person and focus on more than the impact of weight loss on physical health. In addition, the study of new onset depression and/or anxiety during pregnancy is a novel topic. While post-partum depression has been studied extensively, our study focused on depression and anxiety during the perinatal period, not just post-partum.

## Conclusions

This study examined the impact of pre-natal weight loss of 10% or more on mental health outcomes in the perinatal period. The results of the current study suggest that perinatal anxiety is a concern for white mothers and is less likely be a concern for Black mothers. This also suggests that while weight loss prior to pregnancy may have beneficial effects on the physical health of mothers, there is a need to assess how mental health is affected during that time.

## Figures and Tables

**Table 1:** Sample Characteristics Overall and by 10% Weight Loss

	Overall (n = 6085)	No weight loss (n = 5485)	Successful weight loss (n = 600)	p-value
Age (mean, SE)	28.4 (0.06)	28.4 (0.07)	28.7 (0.19)	0.0536
Race	4089 (67.2%)	3605 (65.7%)	484 (80.7%)	< .0001
White	1580 (26.0%)	1489 (27.2%)	91 (15.2%)	
Black	256 (4.2%)	238 (4.3%)	18 (3.0%)	
Latina	160 (2.6%)	153 (2.8%)	7 (1.2%)	
Other				
Depression, prenatal	308 (5.1%)	275 (5.0%)	33 (5.5%)	0.6059
Depression, postpartum	272 (4.5%)	235 (4.3%)	37 (6.2%)	0.0341
Anxiety, prenatal	276 (4.5%)	259 (4.7%)	17 (2.8%)	0.0348
Anxiety, postpartum	311 (5.1%)	278 (5.1%)	33 (5.5%)	0.6485

**Table 2:** Key Variables by Race

	White	Black	Latina	Other	p-value
10% Wt Loss	484 (11.8%)	91 (5.8%)	18 (7.0%)	7 (4.4%)	< .0001
Depression, prenatal	193 (4.7%)	97 (6.1%)	12 (4.7%)	6 (3.8%)	0.1411
Depression, postpartum	197 (4.8%)	61 (3.9%)	10 (3.9%)	4 (2.5%)	0.2430
Anxiety, prenatal	210 (5.1%)	48 (3.0%)	14 (5.5%)	4 (2.5%)	0.0035
Anxiety, postpartum	241 (5.9%)	52 (3.3%)	13 (5.1%)	5 (3.1%)	0.0006

Note. Wt = weight loss.

**Table 3:** Logistic Regression Results for Post-Partum Depression

	Model 1. Unadjusted	Model 2. Adjust for Demographics
10% Wt Loss	1.47 (1.03–2.10)	1.43 (1.00–2.04)*
Age		0.98 (0.96–1.01)
Race		ref
White		0.81 (0.60–1.08)
Black		0.81 (0.42–1.55)
Latina		0.52 (0.19–1.41)
Other		

Note.

* p = 0.0526.

Wt = weight.

**Table 4:** Logistic Regression Results for Prenatal Anxiety

	Model 1. Unadjusted	Model 2. Adjust for Demographics
10% Wt Loss	0.59 (0.36–0.97)	0.54 (0.33–0.90)
Age		1.02 (1.00–1.05)*
Race		ref
White		0.57 (0.41–0.78)
Black		1.06 (0.61–1.85)
Latina		0.46 (0.17–1.25)
Other		

Note.

* p = 0.0614.

Wt = weight

## Data Availability

The data that support the findings of this study are available from the AHEAD Institute at the Saint Louis University but restrictions apply to the availability of these data, which were used under license for the current study, and so are not publicly available. Data are however available from the authors upon reasonable request and with permission of AHEAD Institute. Inquiries into data requests can be sent to ahead@health.slu.edu.

## Abbreviations

BMI: Body Mass Index
VDW: Virtual Data Warehouse
CPT: Current procedural terminology
